# Reciprocal role of cyclins and cyclin kinase inhibitor p21^WAF1/CIP1 ^on lymphocyte proliferation, allo-immune activation and inflammation

**DOI:** 10.1186/1471-2172-6-22

**Published:** 2005-09-21

**Authors:** Ashwani K Khanna

**Affiliations:** 1 Department of Medicine (Nephrology), Medical College of Wisconsin, Milwaukee WI-53226 USA

## Abstract

**Background:**

Immune activation that results due to the aberrant proliferation of lymphocytes leads to inflammation and graft rejection in organ transplant recipients. We hypothesize that the cell cycle control and inflammation are parallel events, inhibition of cellular proliferation by cyclin kinase inhibitor specifically p21 will limit inflammation and prevent allograft rejection.

**Methods:**

We performed in *vitro *and *in vivo *studies using lymphocytes, and rat heart transplant model to understand the role of cyclins and p21 on mitogen and allo-induced lymphocyte activation and inflammation. Lymphocyte proliferation was studied by ^3^H-thymidine uptake assay and mRNA expression was studied RT-PCR.

**Results:**

Activation of allo- and mitogen stimulated lymphocytes resulted in increased expression of cyclins, IL-2 and pro-inflammatory cytokines, which was inhibited by cyclosporine. The over-expression of p21 prolonged graft survival in a completely mismatched rat heart transplant model resulted by inhibiting circulating and intra-graft expression of proinflammatory cytokines.

**Conclusion:**

Cyclins play a significant role in transplant-induced immune activation and p21 over-expression has potential to inhibit T cell activation and inflammation. The results from this study will permit the design of alternate strategies by controlling cell cycle progression to achieve immunosuppression in transplantation.

## Background

Alloimmune activation, caused by aberrant T lymphocyte proliferation is one of the key post transplant events in organ transplant recipients. Current immunosuppressive drugs are therefore designed to inhibit T lymphocyte proliferation. Our previous studies have demonstrated that immunosuppressive drugs, cyclosporine (CsA) tacrolimus (TAC), and sirolimus (SRL) besides inhibiting lymphocyte proliferation and IL-2 also induce the expression of TGF-β and other fibrogenic molecules [1-3] leading to nephrotoxicity and chronic rejection. Therefore, there is a need to develop alternate strategies to achieve immunosuppression for increased graft survival with least nephrotoxicity. The most effective immunosuppression can be achieved by the direct inhibition of T lymphocyte proliferation. Since the expression of cyclins and cyclin-dependent kinases and pro-inflammatory cytokines is increased during T lymphocyte proliferation (4), control of T cell proliferation by regulating the expression of cyclins would potentially inhibit allo-immune activation and inflammation. p21WAF1/CIP1 is one of the most potent cyclin kinase inhibitor and therefore has potential to control the expression of cyclins and T cell activation.

We have demonstrated that CsA, TAC and SRL [4-6] induces the expression of cyclin kinase inhibitor p21WAF1/CIP1 and also *in vitro *and *in vivo *over-expression of p21WAF1/CIP1 in lymphocytes results in decreased response to mitogenic stimuli and greater sensitive to the inhibitory effects of cyclosporine [7]. The present study was designed to study the expression of cyclins during T cell activation, allograft rejection, and the effect of p21WAF1/CIP1 on mitogenic and allogeneic stimulation, pro-inflammatory cytokines and graft survival in a rat heart transplant model.

## Methods

### Preparation of lymphocytes and experimental protocol

Lymphocytes from the normal individuals (n = 4, obtained from Blood Center of Greater Milwaukee, Milwaukee) were separated as described [1]. For mRNA studies, lymphocytes (1 × 10^6^/ml) were cultured with PHA (2 μg/ml), with and without CsA (100 mg/ml) for 4 h, cells were washed twice with PBS and the cells were stored in Trizol at -80 C for RNA preparation.

### Rat cardiac transplantation

Hetrotopic heart transplants were performed as described by us [8]. We used Lewis (LEW, RT1^1^) and Wistar-Furth (WF RT1^u^) rats, which represent complete genetic disparity at both major and minor histocompatibility loci. Isografts were performed in LEW-LEW while allogeneic transplantations were performed in WF-LEW transplant combinations. Immunosuppression was accomplished using CsA at a dosage of 2.5 mg/kg for the whole duration of the experiment described. Rats were monitored daily for evidence of allograft slowing and failure and at the time of rejection, animal s were sacrificed and spleens were used to prepare lymphocytes and organs were snap frozen in liquid nitrogen and stored at -80 C till isolation of RNA.

### Detection of mRNA by reverse transcription and polymerase chain reaction (PCR) in allografts and lymphocytes

Total RNA was isolated from lymphocytes with Trizol (Invitrogen, Carlsbad, CA) and tissues with SV RNA isolation kit (Promega, Madison, WI). Purity of RNA was confirmed by a ratio of 260/280 nm. 1 μg of RNA was reverse transcribed into cDNA using a superscript reverse transcription kit from Invitrogen (Carlsbad CA). The amplification of specific mRNA expression was achieved by polymerase chain reaction (PCR) using specific primer sequences for p21WAF1/CIP1, β-actin, IL-2, TNF-α; Cyclin G, Cyclin E, Cyclin D3, IL-6, and IL-10 are described by us [4-6]. The PCR products were resolved in 1% agarose gel electrophoresis, ethidium bromide stained specific bands were visualized under UV light and photographed. The densitometric analysis of specific bands was made using Alpha-Imager (Alpha Innotech Corp, San Leandro, CA) and data are represented as the ratio of the specific gene to β-actin. We performed cycle analysis for each primer pair to select a cycle number for amplification for each gene studied.

### Jurkat T cells proliferation assay

Cell proliferation was determined using ^3^H-thymidine incorporation as previously described (1–2). All assays were performed in triplicate. A total of 3 individual experiments investigating the proliferation of unaltered and p21WAF1/CIP1 Jurkat cells [described in ref. 7] were performed in unstimulated and activated with PHA. Briefly, 200,000 cells were added to each well of a round bottom 96-well plate. PHA (2 μg/ml) was added to the wells, controls were without PHA. The cells were cultured for 64 h at 37°C in 95% air and 5% CO_2 _enriched environment. The cultures were pulsed with ^3^H Thymidine (1 μCi/well) for the last 16 h of incubation, cells were harvested and radioactivity counted using a scintillation counter. ^3^H -Thymidine uptake was expressed as the mean counts per minute of triplicate samples. The magnitude of Jurkat T cells proliferation from unaltered and p21WAF1/CIP1-augmented cells were investigated at rest following mitogen stimulation.

### *In vivo *transfection of p21WAF1/CIP1

#### p21 sense plasmid DNA

Plasmid DNA was isolated from competent E-coli cells transformed with either the empty pcDNA3.1/Zeo vector (Invitrogen, Carlsbad CA) or the vector containing the full-length p21WAF1/CIP1 gene in the sense direction described by us [7].

### Mixed Lymphocyte Reaction (MLR)

MLR was performed with splenocytes from isografts, untreated and CsA treated transplant recipients (LEW) as responders and irradiated splenocytes from donor strain (WF) as stimulators. The stimulator or responder cells were cultured alone as negative controls. ^3^H Thymidine uptake was expressed as the median counts per minute of triplicate samples. The extent of proliferation determined the allo-reactivity of among these groups of mice and rats.

#### Data analysis

Differences between groups were determined using two-tailed unpaired T test with significance considered present at a p value of less than 0.05. Statistical analysis was performed using a software program from GraphPad Software, Inc., San Diego, CA 92121 USA. The results are expressed as M ± SEM.

## Results

### Effect of inhibition of lymphocyte proliferation on IL-2, cyclins, TNF-α, IL-6 and p21WAF1/CIP1 mRNA

**Figure 1:**
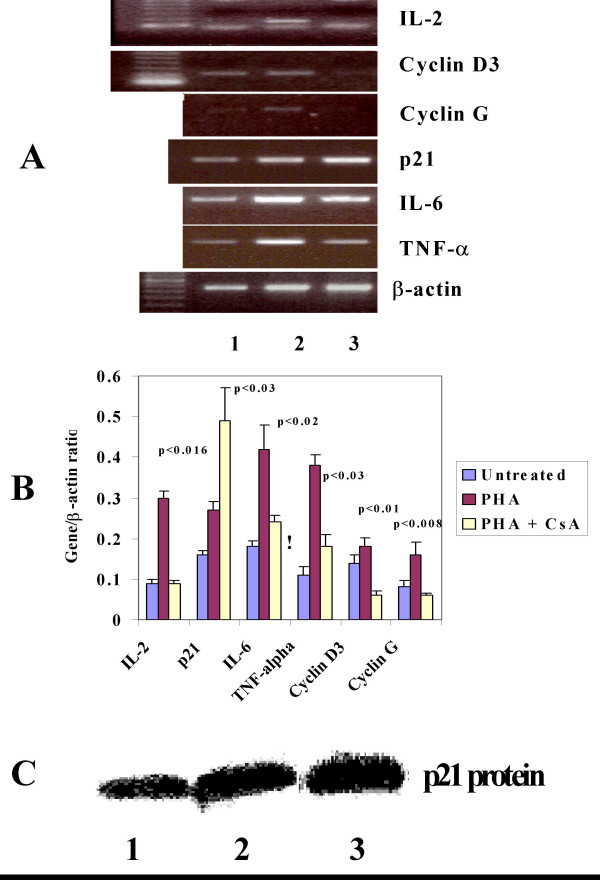
**CsA inhibits cyclins, IL-6 and TNF-α and induces p21WAF1/CIP1 mRNA expression in activated lymphocytes**. **A**). A picture representative of three different experiments demonstrating cyclins (D3 and G), p21WAF1/CIP1, IL-6, TNF-α and induction of mRNA expression in activated lymphocytes (lane 2) with CsA (lanes 3). The lower bands in IL-2 mRNA area are primer dimers. **B**) Mean ± SEM (n = 3) of the ratio of cyclins, IL-6 and TNF-α and induces p21WAF1/CIP1 with β-actin is shown for lymphocytes either unctivated or activated with and without CsA. The p values signify the statistical significance for mRNA expression in lymphocytes activated with and with out CsA for each gene. C) Western blot analysis for p21 protein in lymphocytes unctivated (lane 1), activated (lane 2) with CsA (lane 3).

To understand the relationship between cyclins, pro-inflammatory cytokines and p21WAF1/CIP1 during lymphocyte proliferation and inhibition, we studied the mRNA expression of cyclin G, cyclin D3, IL-2, IL-6, TNF-α, and p21WAF1/CIP1 in lymphocytes activated in the presence or absence of CsA. The expression of IL-2 mRNA was used as a control for activation and inhibition of lymphocytes. The results from a representative of three consecutive experiments are shown in Figure 1A. Mitogen activation of lymphocytes resulted in an increase in IL-2, cyclins G and D3, TNF-α, IL-6 and p21WAF1/CIP1 mRNA (lanes 2), when compared to untreated lymphocytes (lane 1). As expected, CsA inhibited the expression of IL-2, and TNF-α mRNA however IL-6 mRNA was not completely inhibited. More interestingly, whereas the inhibition of lymphocytes by CsA also resulted in a significantly decreased expression of cyclins G, and D3 mRNA, and an increased expression of cyclin kinase inhibitor p21WAF1/CIP1 mRNA (lane 3) was observed.

The results obtained from three consecutive experiments as the ratio of each gene with β-actin (Mean ± SEM) are presented in Figure 1B. A statistically significant decrease in pro-inflammatory cytokines IL-2 (p < 0.016), IL-6 (p < 0.02), TNF-α (p < 0.03) and cyclins; Cyclin D3 (p < 0.01) and Cyclin G (p < 0.008) was observed in sharp contrast to a significant increase in p21 (p < 0.03) in lymphocytes activated in the presence of CsA. CsA treatment also resulted in increased expression of p21 protein in activated lymphocytes (Figure 1C). This increase was about 2 fold.

### Expression of cyclins in lymphocytes from rejecting and non-rejecting rats

**Figure 2:**
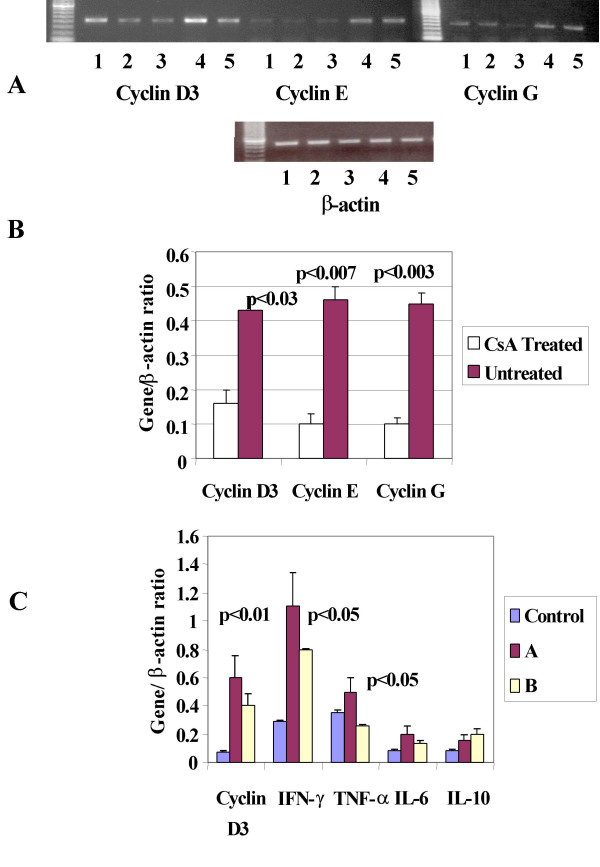
***In vitro *and *in vivo *mRNA expression of Cyclins relates to alloimmune activation**: **A: **Cyclins; D3, E and G and β-actin mRNA expression in lymphocytes isolated from spleens of rat heart transplant recipients. Untreated (lanes 4,5), CsA treated (lanes 1,2,3). **B: **Mean ± SEM of the ratio cyclins with β-actin, p values are calculated between densitometric numbers of untreated and CsA treated transplant recipient. **C: **Cyclin D3 and pro-inflammatory cytokines mRNA expression in lymphocyte from MLR assay using stimulants from donor strain with responders from donor strain (Control) untreated rejecting transplant recipients (A) and CsA treated non rejecting transplant recipients (B). The p values represent the statistical significance between ratio of densitometric numbers for each gene with β-actin from untreated (**Group A**) vs CsA (**Group B**) treated transplant recipients.

The rationale for these experiments is our hypothesis that in untreated and rejecting recipients of cardiac transplants, alloimmune activation will result in an increased expression of mRNA for cyclins in lymphocytes and the treatment with CsA will inhibit alloimmune activation and hence mRNA expression of cyclins leading to the prolongation of graft survival. To test this, we studied the mRNA expression of cyclins G, D3 and E in lymphocytes from rejecting (untreated) and non-rejecting recipients (CsA treated) in a heterotopic rat cardiac allograft model using a fully MHC mismatched [WF (RTl^u^) into LEW (RTl^l^)] strain combination. The results (Figure 2A) demonstrate that the expression of cyclins G, D3 and E was significantly higher in lymphocytes obtained from untreated recipients of cardiac allografts (lanes 4, 5) compared to those treated with CsA (2.5 mg/kg) for 30 days (lanes 1–3). The untreated rats rejected graft between 8–10 days after transplant whereas CsA treated rats did not reject and were sacrificed on day 30^th^. Almost identical expression of the housekeeping gene β-actin in the lymphocytes of these rats is also shown (Figure 2A). The results from the untreated and CsA treated recipients are also presented as the ratio of each cyclin to β-actin (Mean ± SEM); cyclin D3 (0.43 ± 0.05 vs 0.16 ± 0.04, p < 0.03); cyclin G (0.45 ± 0.02 vs 0.1 ± 0.03, p < 0.003) and cyclin E (0.46 ± 0.03 vs 0.1 ± 0.04, p < 0.007). These results (Figure 2B) obtained from a semi-quantitative PCR analysis demonstrate that the aberrant alloimmune activation responsible for graft rejection is associated with the increased expression of cyclins. More significantly, CsA treatment significantly decreased mRNA expression of cyclins, alloimmune activation and prolongation of graft survival.

### Correlation of the expression of cyclins and pro-inflammatory cytokines in allo-immune

To confirm that the increased expression of cyclins in lymphocytes from rejecting rats was due to alloimmune activation, we performed mixed lymphocyte reaction (MLR) using spleen cells from donor animals (WF) as stimulators and splenocytes from recipients (LEW) as responders. Three groups were studied; isografts (control), untreated allografts (A), and CsA-treated allografts (B). After five-day MLR lymphocytes were harvested and washed. RNA was prepared; reverse transcribed to cDNA, and was amplified by RT-PCR for cyclin D3, IFN-γ, TNF-α, IL-6 and IL-10 mRNA. As shown in the Figure 2C the mRNA expression of cyclin D3 was higher in lymphocytes from untreated allografts **group A **as compared to lymphocytes from isografts **group B**. This increased expression of cyclin D3 correlated with the pro-inflammatory cytokines IFN-γ and TNF-α mRNA expression. The expression of IL-6 and IL-10 mRNA was not statistically significant between groups A and B. CsA treatment decreased cyclin D3 expression by 50% and inhibited statistically significant (p < 0.03) IFN-γ mRNA expression. Lymphocyte activation in MLR assay was quantified by ^3^H-thymidine uptake assay. Proliferation of lymphocytes from untreated allografts was significantly higher (two tailed p value = 0.02) compared to CsA- treated allografts (Mean ± SEM of counts per minute, n = 3, 11796 ± 728 vs 7575 ± 360). These results support our conclusions from rat transplant studies that the alloimmune results in increased expression of cyclins mRNA that correlates with production of pro-inflammatory cytokines. Allo-immune activation is demonstrated by increased lymphocyte proliferation from MLR assay, which decreased in CsA treated animals.

### p21WAF1/CIP1 over-expression, lymphocyte proliferation and IL-2 expression

**Figure 3:**
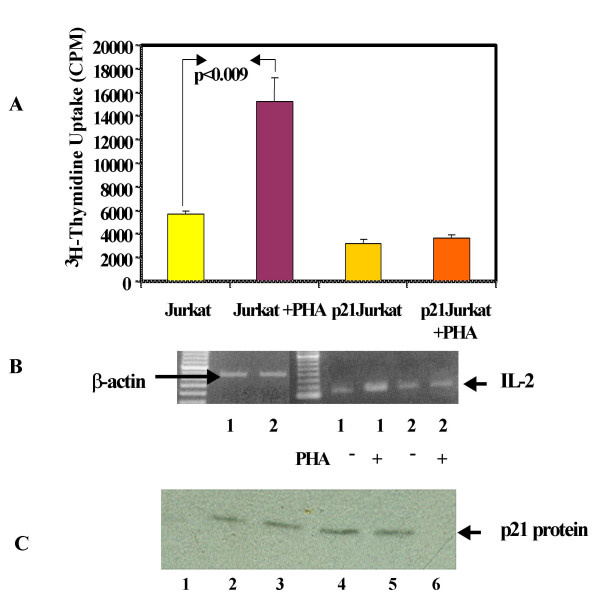
**Over expression of p21WAF1/CIP1 inhibits proliferation and IL-2 mRNA expression in Jurkat T cells**: **A**: A comparison of the proliferation of Jurkat T cells with and without p21 overexpression. **B**. Il-2 mRNA expression in PHA activated normal and p21 over-expressing Jurkat T cells. An identical expression of house keeping gene β-actin is also shown. **C: **p21 protein expression in four different clones of p21 overexpressing Jurkat T cells, Untreated (lane 1), p21 overexpressing (lane 2–5) and transfected with empty vector DNA (lane 6).

To confirm our previous *in vitro *and *in vivo *studies that p21WAF1/CIP1 over-expression will inhibit lymphocyte proliferation and IL-2 expression, we conducted studies using Jurkat T cells. Jurkat T cells were transfected with empty vector plasmid DNA (control DNA) and p21WAF1/CIP1 sense plasmid DNA. We used Jurkat T cells for these experiments because of the ease with which these cells can be transfected as compared to primary T cells; these p21WAF1/CIP1 over-expressing Jurkat T cells are described previously [7]. No differences were observed in the proliferation of normal Jurkat cells and Jurkat cells transfected with empty vector DNA. Control and p21WAF1/CIP1 over-expressing Jurkat T cells were activated with PHA (2 μg/ml) for 4 h for IL-2 mRNA expression studies and 24 h for proliferation studies using ^3^[H]-thymidine uptake assay. The results demonstrate that the control Jurkat cells, but not p21WAF1/CIP1 over-expressing Jurkat T cells, responded to mitogenic stimulation by PHA (Figure 3A). PHA stimulation resulted in an increased expression of IL-2 mRNA in Jurkat cells, not in p21WAF1/CIP1 over-expressing Jurkat cells (Figure 3B). These results indicated that the p21WAF1/CIP1 over-expression rendered Jurkat cells unresponsive to mitogenic stimuli, possibly p21WAF1/CIP1 over-expression did not allow increased cyclin expression, thereby preventing lymphocyte activation by PHA. An increased expression of p21 protein in four different sets of p21-overexpressing Jurkat T cells (lanes 2–4) compared to Jurkat T cells transfected with empty vector plasmid DNA (lane 1) is also shown (Figure 3C) suggesting the increased p21 protein expression in these p21 overexpressing cells.

### Effect of p21WAF1/CIP1 over-expression on graft survival in a rat heart transplant model

#### p21WAF1/CIP1 over-expression in Rats

**Figure 4:**
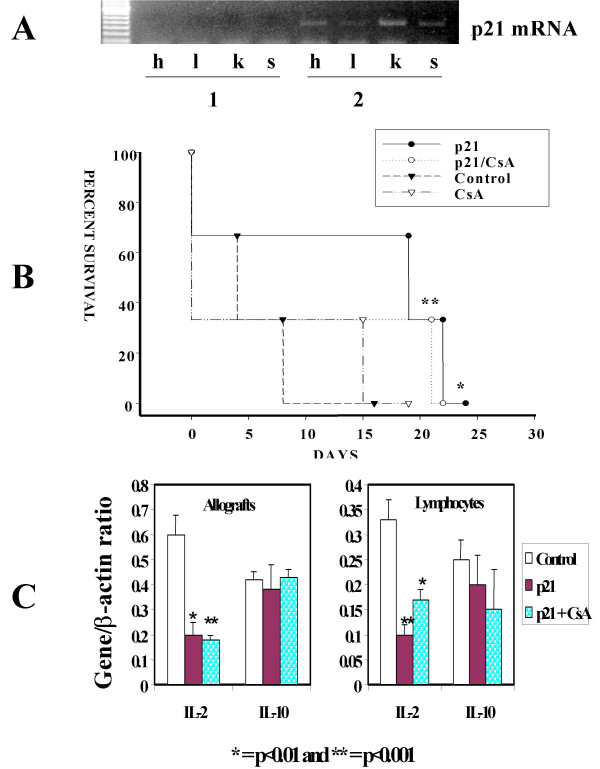
**p21WAF1/CIP1 over-expression prolongs allograft survival**: **A**: p21 Injection of p21 sense plasmid DNA injected mice (set 2not empty vector plasmid DNA induces p21 mRNA expression in heart (h), liver (l), kidney (k) and spleen (s). **B**: Kaplan-Meyer survival graph for rat cardiac transplant recipients. Significant difference in the survival of allografts in p21WAF1/CIP1 transfected recipients compared to controls (* = p < 0.04) and p21WAF1/CIP1 together with CsA (* * = p < 0.005) can be seen. **C**: Effect of p21WAF1/CIP1 over-expression and CsA treatment on mRNA expression of IL-2 in lymphocytes and allografts. A significant decreased expression of IL-2 mRNA expression in lymphocytes and allografts compared to controls is shown (* = p < 0.01) and (* * = p < 0.001).

Encouraged by our studies with mice, which demonstrated that transfection with p21WAF1/CIP1 sense plasmid DNA resulted in decreased lymphocyte proliferation, we performed pilot experiments to determine if *in vivo *over expression of p21 will also result in improved graft survival in a rat cardiac transplant model. We injected (intramuscularly) either p21WAF1/CIP1 sense plasmid DNA or empty vector plasmid DNA (1 mg) to 4 rats in each group. Since in our experiments with mice we used 100 μg of DNA, based on difference in the average weights of a rat and mouse, we used 10 times more DNA in rats. Seven days after the injection, animals were sacrificed, RNA was prepared from heart (h), liver (l), kidney (k) and spleen (s), reverse transcribed to cDNA and amplified for p21WAF1/CIP1 mRNA. Results shown in Figure 4A demonstrate that injection with p21WAF1/CIP1 sense plasmid DNA but not with empty vector plasmid DNA resulted in an over-expression of p21WAF1/CIP1 mRNA. These results also demonstrate that p21WAF1/CIP1 transgenesis using intramuscular injection of plasmid DNA can be achieved in rats. Since during isolation of RNA the contaminating DNA is treated with DNAse, the amplification of injected p21 sense plamsid DNA can be ruled out. The expression of p21WAF1/CIP1 protein using western blot was also detected in spleens, which was the only tissue analyzed (results not shown).

We then studied the effect of modulation of p21WAF1/CIP1 on alloimmunity in a rat heart transplant recipients. We have extensive experience using the completely MHC mismatched WF (RTl^u^) into LEW (RTl^l^) strain combination and have well defined thresholds of cyclosporine-based immunosuppression. In this model, animals reject within 7 to 10 days in the absence of immunosuppression and as late as 180 days with immunosuppression (CsA 2.5 mg/kg). A total of 12 rat transplants divided into four groups (**A-D**) were performed. Recipients in **Group A **were given one intramuscular injection of empty vector plasmid DNA (1 mg); **Group B **rats were given a daily dose of CsA (2.5 mg/kg). Rats in **Group C **rats received three weekly injections of p21WAF1/CIP1 sense plasmid DNA (0.5 mg); and **Group D **rats were given one intra-muscular injection of p21WAF1/CIP1 sense plasmid DNA and a daily injection of CsA (2.5 mg/Kg). The allografts were followed by palpitation, and an arbitrary scale of 1–4 was used to rate the heartbeat to determine the time of graft rejection. The rats were sacrificed when a heartbeat of 1–2 was recorded, which was considered as a cutoff for rejection. Though the number of transplants is low, yet as shown in Figure 4B, p21WAF1/CIP1 alone (*p < 0.04) or in combination with CsA (**p < 0.005) significantly prolonged the graft survival.

To confirm that this effect was due to the inhibition of alloimmune activation, we studied the expression mRNA of IL-2 in lymphocytes isolated from spleens and heart allografts. We also examined the expression of IL-10 mRNA in lymphocytes and allografts. The results are shown in the Figure 4C. The expression of IL-2 mRNA both in lymphocytes and allografts was higher in animals injected with empty vector plasmid DNA demonstrating increased allo-immune activation. IL-2 mRNA expression decreased significantly in recipients treated with p21WAF1/CIP1 sense plasmid DNA alone or together with CsA. The expression of IL-2, correlated with rejection, which indicated an increased immune activity due to allo-immune response resulting in the rejection as compared to p21WAF1/CIP1 or p21WAF1/CIP1 /CsA treated recipients. We did not observe any significant changes in the expression of IL-10 mRNA in allografts, which decreased in animals treated with p21WAF1/CIP1 sense plasmid DNA alone or with CsA, however it did not reach a level of significance (Figure 4C).

## Discussion

The experiments performed in this study were designed to understand the role of cyclins on mitogen and allo-stimulation of immune cells and also, if the inhibition of cyclins will correlate with pro-inflammatory cytokines. We also studied if p21WAF1/CIP1 modulation in recipients of cardiac transplantation modulates allo- and mitogenic stimuli and allograft survival. The results demonstrate that during lymphocyte activation, mRNA expression of cyclins and pro-inflammatory cytokines is significantly increased and CsA inhibited lymphocyte activation, mRNA expression of cyclins, pro inflammatory cytokines but induced p21 mRNA and protein expression.

Studies [9-12] have demonstrated that the expression of cyclin D3, cdk6, and cyclin E is activated in IL-2-stimulated T lymphocytes. However, the novel finding of this present study is that mRNA expression of cyclins in activated lymphocytes correlates with that of pro-inflammatory cytokines, and the expression of both the cyclins and pro-inflammatory cytokines is inhibited by immunosuppressive agent CsA. These are novel findings not demonstrated previously. Our results emphasize that the cell cycle progression and inflammation are concerted events thus regulation of cell cycle control could result in decreased inflammation.

Our *in vitro *findings on the increased expression of cyclins mRNA in activated lymphocytes were reproduced in our *in vivo *studies. The mRNA expressions of cyclin D3, G and E in lymphocytes (possible predominantly T cells, CsA inhibits proliferation of T lymphocytes) isolated from spleens from untreated recipients of rat cardiac transplant were significantly higher than those treated with cyclosporine. The increased expression of cyclins may represent an uncontrolled allo-immune activation in these rats. Since CsA treatment resulted in the inhibition of allo-immune activation and increased graft survival accompanied by a significant inhibition of mRNA expression of cyclins in CsA treated. This possibly was due to the CsA mediated inhibition of alloimmune activation. These results indicate the presence of an active cell cycle progression during allo-immune activation. Therefore, the control of cell cycle progression should prevent inflammation leading to an improved graft survival. These results are supported by our studies with MLR cultures using lymphocytes from rat heart transplant recipients. An increased proliferation of lymphocytes accompanied increased expression of cyclins and pro-inflammatory cytokine mRNA when responders lymphocytes were used from untreated rats as compared to those from isografts or CsA treated rat heart transplant recipients. Again, these activated lymphocytes were possible predominantly T cells, T lymphocyte proliferation is a key component of allo-immune activation. Therefore, these results lend credence to our thinking that the inhibition of allo-immune activation accompanies decreased expression of cyclins and pro-inflammatory cytokines.

These results confirm that control of cell cycle progression plays a significant role in T cell proliferation/activation. Role of p21 in other aspects of lymphocyte proliferation has been studied. Studies of Balomenos et.al, [13] Santiago-Raber et al [14] and Brian et al [15] demonstrated that T lymphocytes from p21WAF1/CIP1^-/- ^mice proliferated significantly more than from wild type mice upon stimulation. These results support our studies that p21WAF1/CIP1 modulation alters cell cycle progression and the immune system. Jackson et al [16] showed that increased levels of p21WAF1/CIP1 at the end of G (1) could prevent cdk-mediated entry into S phase, leading to proliferative unresponsiveness also found in our experiments with p21WAF1/CIP1 over-expressing Jurkat T cells.

The results from this study are of significance because p21 is one of the most potent regulators of the cell cycle and is known to inhibit cell proliferation in two different ways. p21 binds to Cdk2 and inhibits PCNA (proliferating cell nuclear antigen), which is an auxiliary protein in DNA polymerase needed for DNA synthesis and nucleotide excise-n- repair [17]. PCNA has 6 binding sites for p21 [18]. Studies also [19] demonstrated that the PCNA binding and inhibitory activities reside in the C-terminal domain of p21, compared to the location of the CDK inhibitory activity in the conserved N-terminal domain. The authors also concluded that the CDK and PCNA inhibitory domains prevented DNA replication suggested a dual function of p21 as a cell-cycle inhibitor in vivo. We conducted these studies exclusively with cyclin kinase inhibitor p21WAF1/CIP1, though p53 and cyclin kinase inhibitors (p27, p16) have been shown to inhibit cell cycle yet p16 and p21WAF1/CIP1 inhibit cell cycle progression through distinct mechanisms [20]. The specific target for p16 is the Cdk/4cyclin D complex and in a tumor model, p21WAF1/CIP1 and p16 did not show additive or synergistic effects [21]. Furthermore in contrast to p21WAF1/CIP1, the expression of p27 is not under transcriptional control and its mRNA expression remains unchanged during cell cycle [22]. Also, high levels of p27 but not p21WAF1/CIP1 are observed in most quiescent cells and the inhibition of p27 levels precedes the progression of cell cycle [23]. Though both p21WAF1/CIP1 and p27 are critical in the response of cells to mitogens, p21WAF1/CIP1 provides a better balance between cyclins and cyclin kinase inhibitors [24] stressing its significance in inhibition of proliferation/immunosuppression. It is therefore possible that p21WAF1/CIP1 over-expression could interrupt the cell cycle progress and also prevent inflammation. It is well known that during T cell activation, expression of pro-inflammatory cytokines IFN-γ, TNF-α and IL-6 is significantly increased. Since T cells are the key mediators of allo-immune activation, this increased expression of cytokines in organ transplant recipient results in graft rejection [25-27]. Our results demonstrate a parallel increase in the expression of cyclins and pro-inflammatory cytokines. Therefore an inhibition/regulation of cell cycle progression of immune cells by over-expression of cyclin kinase inhibitor p21WAF1/CIP1 would decrease both allo-immune activation and inflammation in transplant recipients.

We also demonstrate that rats transfected with p21WAF1/CIP1 plasmid DNA over expressed p21WAF1/CIP1 mRNA in different tissues. The recipients of cardiac allograft animals who received intra-muscular injection of p21WAF1/CIP1 sense plasmid DNA had significantly increased graft survival compared to the recipients transfected with empty plasmid DNA. These very preliminary studies suggest that p21 overexpression can prolong graft survival to a degree comparable to prolongation by CsA. Further studies will surely be required to confirm and quantify the effect of p21 on graft survival. We also present unique results that the expression of IL-2 mRNA was significantly decreased in both lymphocytes and allografts isolated from p21WAF1/CIP1 over-expressing recipients of rat heart transplants.

Our method of using plasmid DNA to obtain *in vitro *and *in vivo *transfection of p21WAF1/CIP1 is based on the data supporting the efficacy of intra-muscular injection of plasmid DNA for a number of genes [28]. A number of studies [29-32] have demonstrated that non-viral plasmid DNA provides a simple, safe, and viable alternative for gene therapy involving muscle tissue resulting in high level of expression. More significantly plasmids do not induce neutralizing immunity, which permits repeated administration. Rauh et al [33] tested the hypothesis that intramuscular injection of naked DNA could result in the distribution remote from the site of needle placement, facilitating intramuscular gene transfer. Using transcutaneous ultrasound imaging the authors demonstrated that a solution of plasmid DNA administered by direct intramuscular injection into the skeletal muscles of the limb is distributed well beyond the site of needle entry and persisted for 8–10 weeks. Therefore, based on these and our own studies [7], we believe that the p21WAF1/CIP1 over-expression can be obtained through intramuscular injections of plasmid DNA, which could result in the decreased responsiveness of T lymphocytes to allo-and mitogenic stimuli.

In summary, the results from this study uniquely demonstrate that during lymphocyte activation, expression of cyclins is increased and the inhibition of lymphocyte activation by cyclosporine inhibits the expression of cyclins and increases the expression of cyclin kinase inhibitor p21WAF1/CIP1. The expression of cyclins correlates with that of pro-inflammatory cytokines like TNF-α and IFN-γ *in vitro *in activated lymphocytes and *in vivo *in lymphocytes from animals with rejecting rat heart transplants. These studies demonstrate that cyclins and pro-inflammatory cytokines are key mediators of allo-immune activation and the alteration of p21WAF1/CIP1 expression can modulate lymphocyte proliferation and allo-immune activation. These studies uniquely provide evidence on the role of cell cycle control molecules on allo-immune activation and will allow the development of alternate strategies to obtain improved graft survival in organ transplantation. Moreover based on our previously published studies, the results presented in this study, and our recently published study [34] we believe that p21WAF1/CIP1 might provide better immunosuppression with least side effects observed with the currently clinically used immunosuppressive drugs for the organ transplant recipients.
